# First Year of Israeli Newborn Screening for Severe Combined Immunodeficiency—Clinical Achievements and Insights

**DOI:** 10.3389/fimmu.2017.01448

**Published:** 2017-11-06

**Authors:** Erez Rechavi, Atar Lev, Amos J. Simon, Tali Stauber, Suha Daas, Talia Saraf-Levy, Arnon Broides, Amit Nahum, Nufar Marcus, Suhair Hanna, Polina Stepensky, Ori Toker, Ilan Dalal, Amos Etzioni, Shlomo Almashanu, Raz Somech

**Affiliations:** ^1^Pediatric Department A and the Immunology Service, Jeffrey Modell Foundation Center, Edmond and Lily Safra Children’s Hospital, Sheba Medical Center, Affiliated to the Sackler Faculty of Medicine, Tel Aviv University, Tel Aviv, Israel; ^2^The National Center for Newborn Screening, Israel Ministry of Health, Tel-HaShomer, Israel; ^3^Pediatric Immunology Clinic, Soroka University Medical Center, Faculty of Health Sciences, Ben-Gurion University of the Negev, Beer Sheva, Israel; ^4^The Jeffrey Modell Foundation Israeli Network for Primary Immunodeficiency, New York, NY, United States; ^5^Allergy and Immunology Unit, Schneider Children’s Medical Center of Israel, Felsenstein Medical Research Center, Petach Tikva, Sackler Faculty of Medicine, Tel Aviv University, Tel Aviv, Israel; ^6^Ruth Children Hospital, Rappaport Faculty of Medicine, Technion – Israel Institute of Technology, Haifa, Israel; ^7^Bone Marrow Transplantation Department, Hadassah Hebrew University Medical Center, Hadassah-Hebrew University Medical School, Jerusalem, Israel; ^8^Allergy and Clinical Immunology Clinic, Department of Pediatrics, Shaare Zedek Medical Center, Hadassah-Hebrew University Medical School, Jerusalem, Israel; ^9^Pediatric Allergy Unit, Wolfson Medical Center, Holon, Israel; ^10^Pediatric Department, Wolfson Medical Center, Holon, Israel; ^11^Sackler Faculty of Medicine, Tel Aviv University, Tel Aviv, Israel; ^12^The National Lab for Diagnosing SCID – The Israeli Newborn Screening Program, Israel Ministry of Health, Tel-Hashomer, Israel

**Keywords:** severe combined immunodeficiency, newborn screening, T cell development, preterm, immunodeficiency

## Abstract

Severe combined immunodeficiency (SCID), the most severe form of T cell immunodeficiency, is detectable through quantification of T cell receptor excision circles (TRECs) in dried blood spots obtained at birth. Herein, we describe the results of the first year of the Israeli SCID newborn screening (NBS) program. This important, life-saving screening test is available at no cost for every newborn in Israel. Eight SCID patients were diagnosed through the NBS program in its first year, revealing an incidence of 1:22,500 births in the Israeli population. Consanguine marriages and Muslim ethnic origin were found to be a risk factor in affected newborns, and a founder effect was detected for both *IL7Rα* and *DCLRE1C* deficiency SCID. Lymphocyte subset analysis and TREC quantification in the peripheral blood appear to be sufficient for confirmation of typical and leaky SCID and ruling out false positive (FP) results. Detection of secondary targets (infants with non-SCID lymphopenia) did not significantly affect the management or outcomes of these infants in our cohort. In the general, non-immunodeficient population, TREC rises along with gestational age and birth weight, and is significantly higher in females and the firstborn of twin pairs. Low TREC correlates with both gestational age and birth weight in extremely premature newborns. Additionally, the rate of TREC increase per week consistently accelerates with gestational age. Together, these findings mandate a lower cutoff or a more lenient screening algorithm for extremely premature infants, in order to reduce the high rate of FPs within this group. A significant surge in TREC values was observed between 28 and 30 weeks of gestation, where median TREC copy numbers rise by 50% over 2 weeks. These findings suggest a maturational step in T cell development around week 29 gestation, and imply moderate to late preterms should be screened with the same cutoff as term infants. The SCID NBS program is still in its infancy, but is already bearing fruit in the early detection and improved outcomes of children with SCID in Israel and other countries.

## Introduction

The purpose of newborn screening (NBS) is to identify newborns that are at risk for a panel of metabolic, endocrine, or hematologic diseases in which early diagnosis and prompt treatment will dramatically change disease outcome of affected patients (1). One such disease that has been recently added to NBS panels worldwide, is severe combined immunodeficiency (SCID), the most profound inherited immunodeficiency (2). SCID encompasses a heterogeneous group of genetic disorders manifest by increased susceptibility to life-threatening and opportunistic infections at early life and is characterized by arrest of T lymphocyte maturation and variable alterations in B and natural killer (NK) cells differentiation (3). It was shown that SCID infants diagnosed and treated with hematopoietic stem cell transplantation in the first 3.5 months of life had improved survival and reduced morbidity (4). This observation has made SCID a very attractive target for NBS (5). Screening for SCID is of value particularly in communities, such as in Israel, where a relatively high frequency of consanguineous marriages is known to exist (6). While the accepted frequency of SCID in most western countries is 1:58,000 births, it is estimated that in such communities the frequency of SCID will be higher (7–10). SCID can be detected early in life with the use of T cell receptor excision circle (TREC) quantification on dry blood spots obtained from a Guthrie card (11). TREC, a DNA marker that is formed as a byproduct during the normal process of T cell receptor (TCR) development, is a highly sensitive and specific tool to estimate T cell immunity in any medical conditions where T cells are known to be affected (12). For SCID detection, it can obviously be used in patients with no T cells but can also identify patients with clonally expended T cells or with a limited TCR repertoire (13). Normal production of TREC begins in early embryonic development, around 13 weeks of gestation, and rises progressively throughout pregnancy, reaching decent and easy to detect levels at birth (14). TREC quantification has been established as the most useful and inexpensive high-throughput assay to screen newborns for SCID (15, 16). This screening test is currently being used successfully across the US and in several additional countries (8, 17–19). The primary target of the screening is to identify SCID patients. As a secondary target, the screening identifies other causes of T cell immunodeficiencies including specific syndromes, severe prematurity, secondary T cell lymphopenia or undefined causes of T cell lymphopenia (20). A recent successful pilot study that was conducted by us was able to retrospectively identify seven Israeli patients with SCID (13). This paved the way toward adding NBS for SCID to the national NBS program by the Israeli Ministry of Health (21). As of October 1, 2015, this test is available at no cost for all newborns in Israel and the confirmatory process is performed in one center. Here, we summarize our experience with 1 year of NBS for SCID and report several perspectives regarding T cell development in non-immunodeficient newborns that emerged from the accumulated data.

## Materials and Methods

### Population

Data comprised of TREC results and accompanying epidemiologic parameters (sex, gestational age, birth weight, singleton/twins/triplets) for newborns born in Israel between October 1, 2015 and September 30, 2016, drawn from the computer archives of the Israeli National Center for NBS. Data entries with missing information or containing apparent typing errors were removed from analysis, as were entries for samples with poor DNA amplification (beta-actin <16 copies). Additionally, gestational age/birth weight norms were calculated and newborns for which birth weight was above 5 SD or below −5 SD for their respective gestational age were removed from analysis (*n* = 93). Finally, analysis was performed for 177,277 newborns.

The primary target of the screening program for newborns was to identify those with SCID (defined by us as less than 300/μl CD3^+^ T cells in peripheral blood) or leaky SCID (T lymphopenia but >300/μl CD3^+^ T cells). The secondary target was to identify newborns with non-SCID lymphopenia (classified into groups as syndromic patients, lymphopenia due to secondary causes, prematurity, or unknown etiology). False positives (FPs) were defined as newborns with consecutive positive screening results, whose clinical presentation was unremarkable and immunological workup was negative for lymphopenia of any etiology.

The study was approved by both the Sheba Medical Center institutional review board as well as the Israeli national research committee. Informed consent was obtained from all individual participants included in the study where genetic evaluation was needed.

### Specimen Testing

The Israeli SCID NBS program uses the commercial EnLiteTM Neonatal TREC kit (Wallac Oy, Mustionkatu 6, FI-20750 Turku, Finland). Briefly, Dried Blood Spot (DBS) punches of 1.5 mm diameter are inserted into a black, 96-well PCR plate. DNA is eluted without extraction. Next, PCR amplification of TREC and beta-actin, an internal control for each specimen, is performed. Four PCR plates are processed in parallel, each plate containing a standard TREC curve in triplicates, as well as positive and negative controls for both targets.

### Mutation Analyses

Severe combined immunodeficiency mutations were identified using either Whole Exome Sequencing (WES) or direct Sanger sequencing (where family history allowed targeted sequencing of suspected genes). Results were than validated through Sanger sequencing of patients’ parents, healthy siblings, or both.

### Lymphocyte Subset Determination

Cell surface markers of peripheral blood mononuclear cells (PBMCs) were determined by immunofluorescent staining and flow cytometry (Navios, Beckman Coulter, Brea, CA, USA) using anti-CD3, anti-CD19, anti-CD16, and anti-CD56 from BD Biosciences; and anti-CD4 and anti-CD8 from Beckman Coulter.

### Proliferation Assay

T cell proliferation was tested by standard H^3^-thymidine uptake assays (1 μCi/well) by culturing 10^5^ PBMCs with phytohemagglutinin (1 µg/ml; Sigma-Aldrich) or plastic bound anti-CD3 (10 µg/ml UCHT-1 from BD) for 72 h.

### TCR Repertoire Analysis

Surface expression of individual TCRVβ families was analyzed using flow cytometry and a set of Vβ-specific fluorochrome-labeled monoclonal antibodies as previously described (22). Normal control values were obtained from the IOTest Beta Mark-Quick Reference Card (Beckman Coulter).

### Quantification of TREC in Peripheral Blood

T cell receptor excision circle copy numbers were determined using quantitative real-time PCR (qRQ-PCR). PCRs were performed as previously described (22) using as template 0.5-µg genomic DNA (gDNA) extracted from PBMCs. RQ-PCR was carried out using an ABI PRISM 7900 Sequence Detector System (Applied Biosystems). A standard curve was constructed by using serial dilutions containing 10^3^–10^6^ copies of a plasmid with known TREC copy numbers. Patient and control samples were tested in triplicate, and the number of TRECs in a given sample was calculated by comparing the obtained cycle threshold value of the sample to the standard curve using an absolute quantification algorithm.

### Statistical Analyses

Statistical analyses were performed using SPSS software (IBM Corp. Released 2016. IBM SPSS Statistics for Windows, Version 24.0. Armonk, NY, USA: IBM Corp.). Given the skewed distribution of the data, the Mann–Whitney *U* test and the Kruskal–Wallis test were used to compare continuous variables between groups. Correlation between continuous variables was assessed using the Spearman’s rank correlation coefficient. All statistical tests were two-tailed. Differences were considered statistically significant when the *p* value was less than 0.05. Whenever applicable, results were presented as median, with 25th to 75th percentiles in brackets.

## Results

### Screening Overview

A total of 188,162 newborns were screened between October 1, 2015 and September 30, 2016. After subtracting entries with missing data, errors, and poor DNA amplification, data of a total of 177,277 newborns were analyzed. Of these, 51.5% were male. Median TREC copy number/blood spot for all DBS samples was 107 (69–169, 25th to 75th percentiles), median gestational age 39 weeks, median birth weight 3,240 g (2,925–3,545). 12,880 (7.26%) infants were born prematurely (<37 weeks gestation). 11,316 (87.8% of all preterm newborn) were moderate to late preterm (per WHO definition, 32 to <37 weeks), 1,126 (8.7%) very preterm (28 to <32 weeks), and 438 (3.4%) extremely preterm (<28 weeks). 1,614 (0.9%) were born Very Low Birth Weight (VLBW; per WHO definition, weighing ≤1,500 g), 12,293 (6.9%) were born Low Birth Weight (LBW; >1,500 to ≤2,500 g).

T cell receptor excision circle cutoff for retesting was initially set at 36 copies per blood spot and was gradually lowered to 23 copies per blood spot by year’s end. The Israeli SCID screening algorithm and the rate of positive results with different cutoffs are reviewed elsewhere (23). In 561 instances (0.3%), a second Guthrie card was requested following an initial positive result. Forty-six infants (0.02%) were referred to the national center for SCID screening confirmation following consecutive positive results on two separate Guthrie cards.

### Primary Target

The primary target of the screening program was to identify infants with SCID or leaky SCID and to distinguish them from infants with FP screening results. During the first year of the screening program, 8 infants were diagnosed as SCID or leaky SCID (Table 1) and 11 infants received a diagnosis of FP. Consanguine marriage and Arab-Muslim origin were more frequent in the SCID patients (7 of 8) compared to the FP group (1 and 3, respectively). Of note, while consanguineous marriages are relatively high in Israel compared to other developed countries as a whole, the rate of consanguinity is particularly high among Arab-Muslims. Three patients belonged to the same extended family, though not immediately related. Two patients had positive family histories for SCID. The Israeli confirmation protocol consists of TREC measurement in peripheral blood, proliferation in response to mitogen stimuli, and flow cytometry analysis for total lymphocyte profile and the expression of TCRVβ repertoire. Per definition, all confirmatory tests and outcome measurements (growth and development, infections, hospitalization, and overall general appearance) were completely normal in newborns with FP results. All SCID patients had lymphopenia (Table 2). They could be classified as true SCID (5 patients) and leaky SCID (3 patients) based on the number of autologous CD3^+^ T cells (more or less than 300 cells/μl). Four patients had SCID variants with normal B cell counts (B^+^ SCID) and four had SCID variants with decreased B cell counts (B^−^ SCID). All had normal numbers of NK cells. T cell proliferation was relatively normal in three patients and reduced or absent in the rest (3 and 2, respectively). Similarly, assaying TCRVβ repertoire did not prove sufficient for diagnosis as three of the SCID patients had only mildly abnormal results. The remaining patients had either a skewed repertoire (two patients) or the test could not be performed due to absence of T cells (three patients). TREC in DBS, obtained during the NBS, was undetectable in all SCID patients and in 7/11 infants in the FP group. The remaining four FP had an average of 10.4 ± 8.6 copies per blood spot. TREC quantification in peripheral blood was significantly reduced in all SCID patients compared to the FP group (36.8 ± 40.4 vs 1,984.6 ± 1,944.5, normal >400 copies per 0.5 µl DNA). Test sensitivity and specificity were calculated for each confirmation test with regards to its ability to distinguish between SCID patients and all other newborns referred to confirmation testing (FP and secondary targets). TREC in peripheral blood, total lymphocytes, and CD3^+^ are more sensitive and less specific than CD4^+^, TCRVβ, or proliferation assay (Table 3).

**Table 1:** Severe combined immunodeficiency patients clinical and genetic data.

Patient	Major infection	Diagnosis	Mutation	Outcome
P1	None	*DCLRE1C*^a^	c.1299_1306dup-AGGATGCT (homozygous)	A/W, Post-BMT
P2	None	*DCLRE1C*^a^	c.1299_1306dup-AGGATGCT (homozygous)	A/W, Post-BMT
P3	None	*DCLRE1C*^a^	c.1299_1306dup-AGGATGCT (homozygous)	A/W, Post-BMT
P4	None	*IL7R*α^b^	c.120C > G; p. F40L (homozygous)	A/W, No-BMT
P5	None	*DCLRE1C*^c^	del. ex1-3 (homozygous)	A/W, Post-BMT
P6	Yes	*IL7R*α^b^	c.120C > G; p. F40L (homozygous)	A/W, Post-BMT
P7	None	Complete DGS	Unknown	Deceased
P8	None	RMRP^d^	ins.17bp TIS-4 TCTGTGAAGCTGAGGAC TIS + 239 C > T	A/W, Post-BMT

*DGS, DiGeorge syndrome; A/W, alive and well; BMT, bone marrow transplant; RMRP, RNA component of mitochondrial RNA processing endoribonuclease, causative gene for Cartilage-Hair Hypoplasia; TIS, transcription initiation site*.

*^a^Accession no. NM_001033855*.

*^b^Accession no. Chromosome 10, NC_000010*.

*^c^Accession no. NM_002185*.

*^d^Accession no. NG_017041*.

**Table 2:** Severe combined immunodeficiency patients confirmatory tests results.

Patient	DBS TREC^a^	PB TREC^a^	Total Lymphocytes^b^	CD3^**+**b^	CD4^**+**b^	Proliferation^c^ (%)	TCR repertoire
P1	0	74	1,695	1,135	728	100	Normal
P2	0	0	393	35	35	0	Skewed
P3	0	105	1,392	807	626	50	N/D
P4	0	57	2,568	360	257	70	Normal
P5	0	0	1,162	0	70	0	N/D
P6	0	9	924	101	64	30	Normal
P7	0	0	2,000	4	0	0	N/D
P8	0	50	886	88	35	25	Skewed

*^a^Copy numbers per 3 mm blood spot for DBS, per 0.5 µg DNA for peripheral blood*.

*^b^Cells/μl*.

*^c^Proliferation assay results are displayed as percent of healthy age matched control’s result*.

*DBS, dried blood spot; TREC, T cell receptor excision circles; PB, peripheral blood; TCR, T cell receptor; N/D, not done*.

**Table 3:** Statistical measures of confirmatory tests.

Test	Sensitivity (confidence interval)	Specificity (confidence interval)
Total lymphocytes	87.5% (52.9–97.8)	75% (56.6–87.3)
CD3^+^	87.5% (52.9–97.8)	82.1% (64.4–92.1)
CD3^+^CD4^+^	75% (40.9–92.9)	89.3% (72.8–96.3)
Proliferation	62.5% (30.6–86.3)	95.2% (77.3–99.2)
TCR repertoire	62.5% (30.6–86.3)	93.1% (78–98.1)
PB TREC	100% (67.6–100)	72.4% (54.3–85.3)

*PB, peripheral blood; TREC, T cell receptor excision circles; TCR, T cell receptor*.

For seven of the eight SCID patients, a genetic etiology was revealed (Table 1) suggesting a founder genetic effect for both *IL7r*α and *DCLRE1C* (encoding ARTEMIS) SCID. Six of the seven identified mutations were homozygous, whereas P8 was found to harbor compound heterozygote mutations (Table 1). For the eighth patient, though phenotypically consistent with DiGeorge syndrome (typical facies, cardiac defect, hypocalcemia, and severe lymphopenia), a genetic etiology was not identified even with the use of chromosomal microarray (CMA) and WES. Seven of the eight SCID patients detected through the screening program are currently alive, six have already undergone successful bone marrow transplantation (BMT). One patient succumbed to cardiac complications associated with her syndrome. One patient despite having genetically verified SCID, has displayed a spontaneous recovery of her immune system due to yet unknown reasons. No typical SCID patients “missed” by the screening have been reported in Israel since the initiation of the screening program. All infants defined as FPs are currently alive and well, not requiring special medical attention over a follow-up period of at least 1 year.

### Secondary Targets

Clinical assessment and results of confirmation tests identified 27 newborns that were considered to be part of the secondary target of the program. These could be categorized into four groups. Nine newborns were diagnosed with congenital syndromes with variable degrees of T-cell impairment (four with Down syndrome, two with partial DiGeorge syndrome, one with multiple congenital anomalies, two with unknown syndromes). Complete confirmation tests were performed in only five of these syndromic patients. In nine newborns, the abnormal results were attributed to extreme prematurity with slow recovery of the immune system. Complete confirmation tests were available in only five of them. Four patients were diagnosed with secondary T cell immunodeficiency (three cases of chylothorax and one case of an infant born to a mother who was treated with immunosuppressive agents during pregnancy). Finally, five newborns, for which some of confirmation tests were abnormal (thus excluding them as FP) could not be classified. By 1 year, all of these children had normal repeat workup. They required no medical intervention.

### Correlation between Gestational Age and TREC

**Figure 1:**
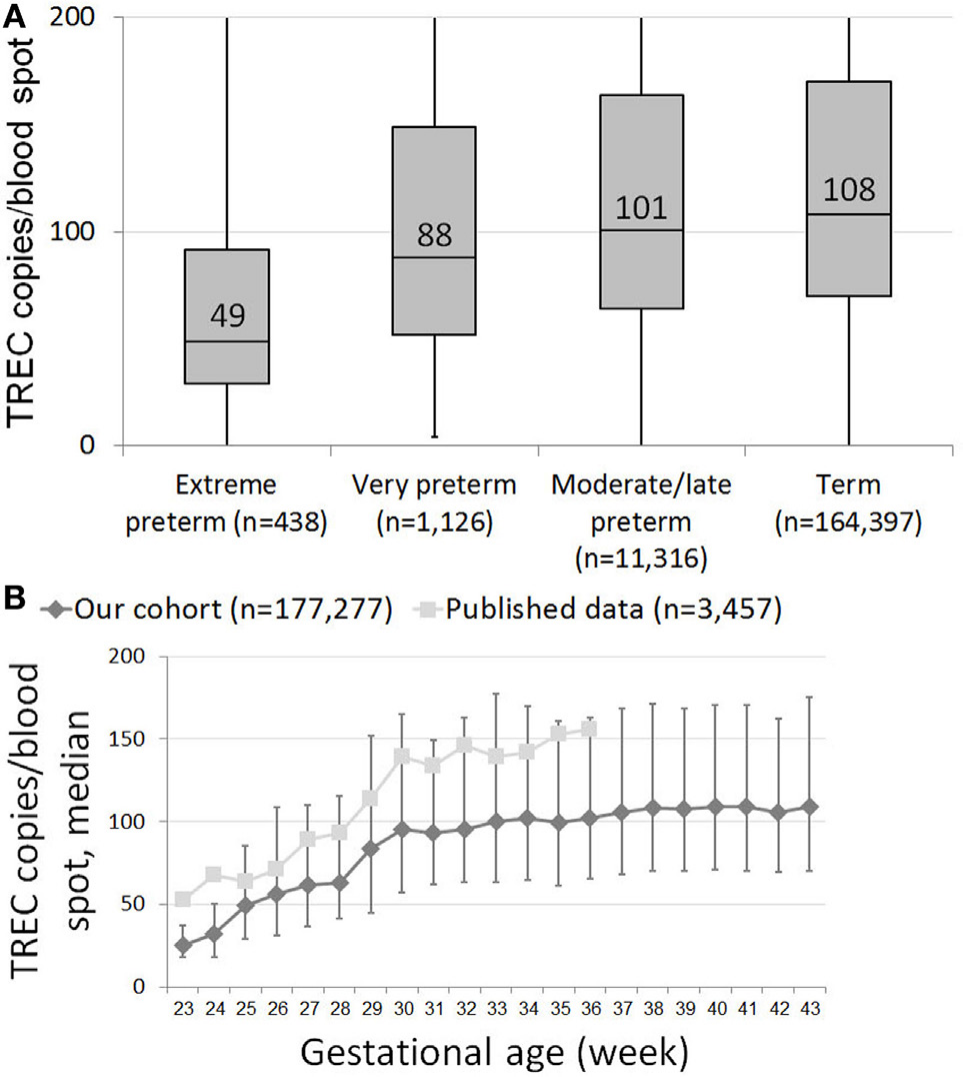
**(A)** Box and whiskers plot showing T cell receptor excision circle (TREC) levels for each gestational age group. Extreme preterms = below 28 GAW (gestational age weeks, *n* = 438); very preterm = 29–32 GAW (*n* = 1,126); moderate/late preterm = 33–36 GAW (11,316); term = 37–45 GAW (*n* = 164,397). **(B)** Median TREC copy numbers/blood spot for each gestational week. Results from our cohort are marked as diamonds, results from Barbaro et al. are marked as squares (18). Error bars indicate 25th and 75th percentile TREC copy numbers for each gestational week.

T cell receptor excision circle copy numbers rose consistently and significantly along with gestational age in our healthy newborn cohort (Figure S1 in Supplementary Material). There was great variability in TREC results within each birth week, resulting in a mild positive correlation (0.256, *p* < 0.001) between gestational age and TREC only in extreme preterms, and no correlation between age and TREC for all other age groups. When looking at median TREC for age groups, due to the large sample size, there was a significant difference in median TREC values between each preterm group and term infants (Figure 1A). However, while the difference between term and moderate to late preterm TREC was significant but not meaningful, 108 (70–170) vs 101 (64–164) median TREC (*p* = 0.0017), the differences between term and extremely preterm, 49 (29–92), or very preterm, 88 (52–149), were both highly significant (*p* = 1E^−16^, *p* = 4E^−6^, respectively) and meaningful. When looking at each birth week separately, one can observe a steady increase in median TREC from week to week between 23 and 28 weeks gestation, followed by a surge in TREC values between 28 and 30 weeks gestation, when median TREC rises by 32 (a 150% increase), another period of incremental increases from week 30 on until reaching a plateau around gestational week 38 (Figure 1B).

### Correlation between Birth Weight and TREC

**Figure 2:**
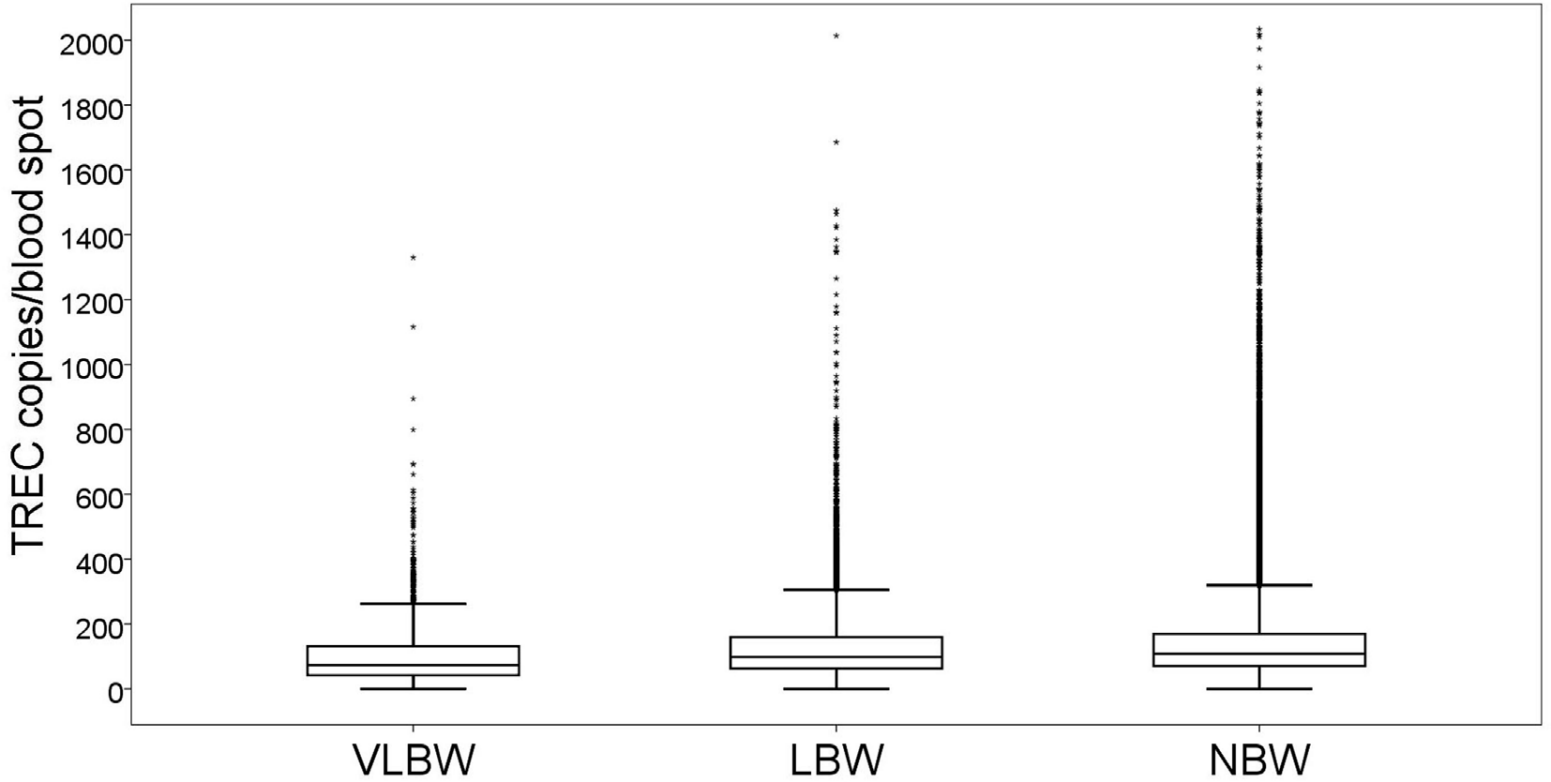
Box and whiskers plot showing T cell receptor excision circle (TREC) levels for each birth weight group (total *n* = 177,277). VLBW = very low birth weight, below 1,500 g; LBW = low birth weight, 1,500–2,500 g; NBW = normal birth weight, above 2,500 g. Box = 25th and 75th percentiles, whiskers = 1.5 times height of box or, if no case has a value in that range, the min/max value, asterisks = outliers.

T cell receptor excision circle copy numbers rose consistently and significantly along with birth weight (Figure 2). Newborns with VLBW had a median TREC of 73.6 (41.8–131), compared to 98.6 (62.9–160) in newborns with LBW and 108.6 (70.2–170) in newborns with NBW. All differences were statistically significant (VLBW vs LBW *p* = 1.7E^−8^, VLBW vs NBW *p* = 5.2E^−19^, LBW vs NBW *p* = 9.8E^−13^). The correlation between birth weight and TREC was more pronounced for extremely premature newborns (0.309) than for newborns of other age groups (0.147 for very premature newborns and no correlation for late preterm and term infants). All correlations were statistically significant, *p* < 0.001.

### Correlation between Sex and TREC

Median TREC for female neonates was 115.2 (74.6–179.7), compared to 100.8 (65.4–158.6) for male neonates. The difference was extremely significant (*p* = 7E^−202^). The groups were similar in terms of gestational age (39.0, 38.9, respectively) and birth weight (3,147 g, 3,268 g, respectively). Correlations between gestational age and TREC, as reported above, were maintained when stratified into female (Figure S2 in Supplementary Material) and male (Figure S3 in Supplementary Material) infants. Correlations between birth weight and TREC, similarly, were maintained when sectioning the data into female (Figure S4 in Supplementary Material) and male (Figure S5 in Supplementary Material) infants.

### TREC Recovery Rate in Preterm Infants

**Figure 3:**
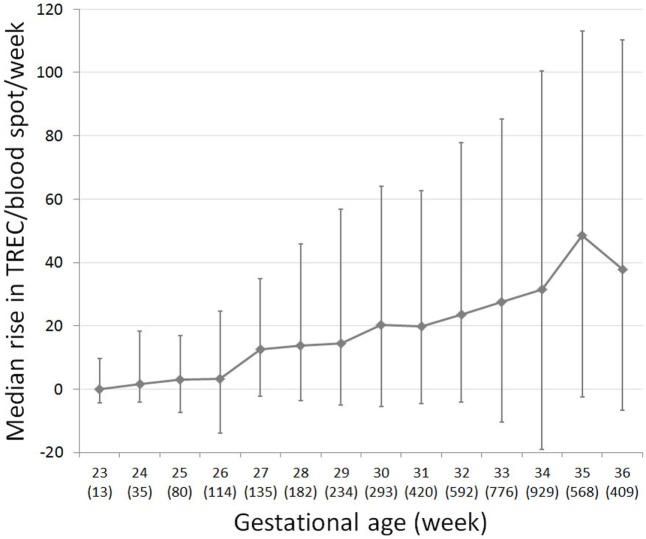
T cell receptor excision circle (TREC) recovery rate in preterm infants, for which multiple TREC results were collected at separate time points (total *n* = 4,780). Median change in TREC copies/blood spot per week for each gestational week. Number of subjects per week listed in brackets below gestational week. Error bars indicate 25th and 75th percentile TREC change for each gestational week.

Per the Israeli SCID screening algorithm, repeat DBS were collected and tested for TREC for preterm infants who remain hospitalized for an extended time period, regardless of their initial TREC result. We examined the rate of TREC increase per week for 4,212 preterm infants, for whom TREC results from multiple time points were available. Individual TREC dynamics were extremely variable. For all preterm infants, the median rate of TREC increase per week was 24 (−7.6 to +79). However, TREC increase rate changes dramatically with birth week (Figure 3). For infants born at 23 weeks gestation, median TREC decreased by 0.07 (−4.2 to +9.6) per week, whereas for infants born at 35 weeks gestation, median TREC increased by 48 (−2.6 to +113) per week. TREC increase rate was very well correlated with gestational age (*R*^2^ = 0.9229). These data are limited by the fact that it pertains to a hospitalized population and may not represent the general population.

### Twins

**Figure 4:**
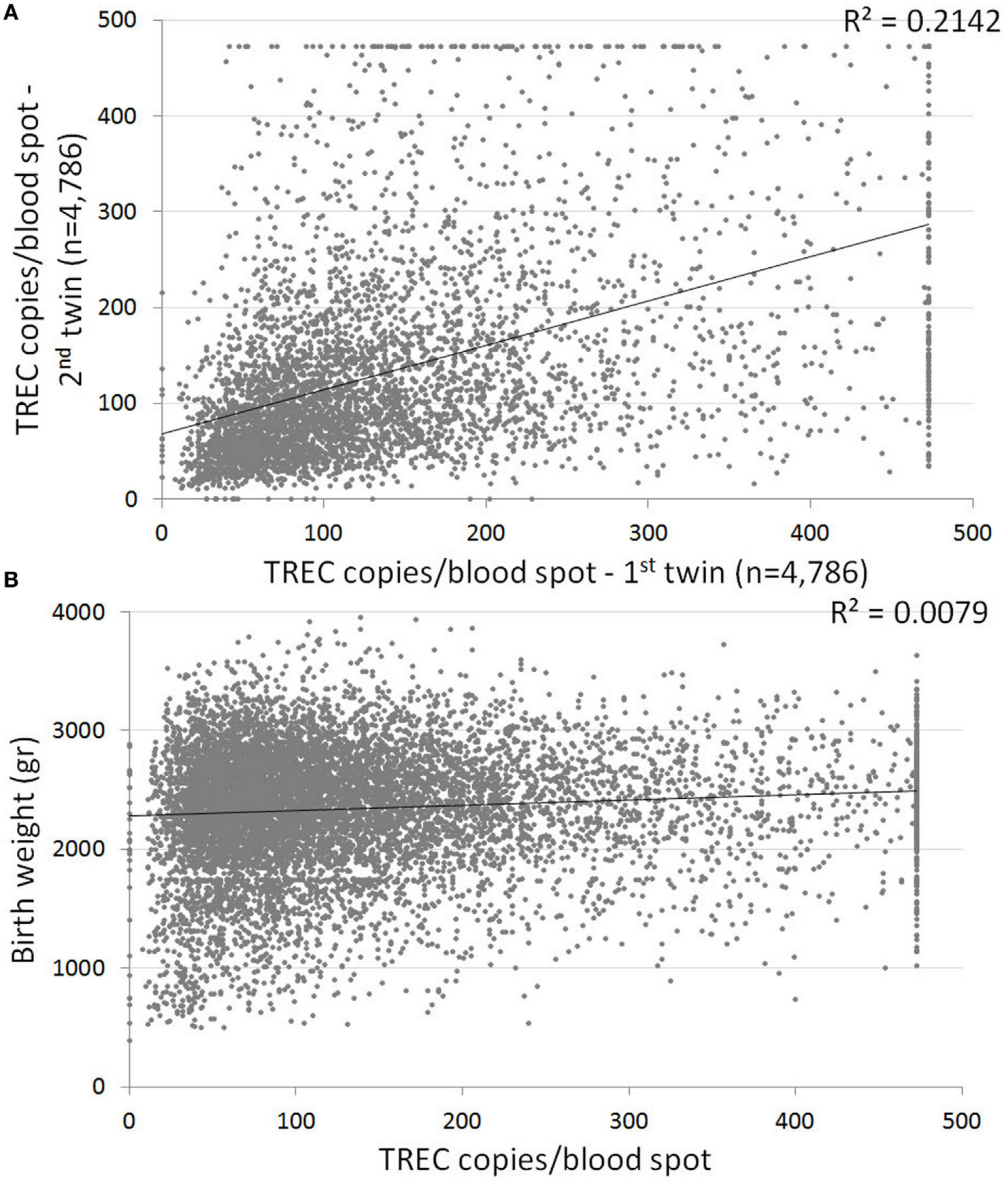
**(A)** Correlation between T cell receptor excision circle (TREC) values of twins. Each dot represents a twin set. Linear trend line indicates a weak correlation between twin TREC results, with a 0.2 coefficient of determination. *n* = 4,786 sets of twins. **(B)** Correlation between birth weight and TREC values for the twin cohort. Linear trend line indicates no correlation (0.0079 coefficient of determination).

Our cohort included 4,786 sets of twins. The average gestational age of all twin pairs was 35.77 weeks; the average birth weight was 2,338 g. The median TREC for all twins was 103 (64–172), significantly lower than the median TREC for singletons, as expected due to lower gestational ages and birth weight. Despite similar average birth weights between twins (2,359 and 2,318, first and second twin accordingly), the first twin in each set had a median TREC of 107 (67–177), compared to only 100 (62–166) for the second twin (*p* = 0.003). The correlation between TREC values of twin sets was low (Figure 4A), highlighting the great inter-individual variability in TREC. The different TREC levels according to birth order appear to be unrelated to differences in birth weight in our cohort, as no correlation was found between birth weight and TREC copies/blood spot in the twin cohort (Figure 4B).

## Discussion

The NBS program for SCID achieves its primary goal, of detecting infants with SCID mere days after birth so that they may receive prompt, disease course altering treatment, and it does so with resounding success (8, 24). However, as any major program in its infancy, there is great potential benefit in fine-tuning the screening algorithm, in order to minimize FP results, better define secondary targets, and glean information about normal and abnormal immune development from an ever growing database of patients and healthy infants.

T cell receptor excision circle analysis has been the method of choice for screening of newborns for severe forms of primary T cell lymphopenia. Our one year experience has reiterated the feasibility of NBS followed by confirmatory tests as a means to successfully identify SCID. Eight SCID patients were identified during the first year of the Israeli NBS program. In the US, NBS for SCID revealed an incidence of 1.72:100,000 births in the general population (8). A similar incidence (~1.69:100,000) was found in a recent meta-analysis, where 13 relevant studies were included (24). Due to high rates of consanguine marriages in Israel, it was assumed the incidence of SCID would be higher, and indeed, according to these data the incidence of SCID in Israel is 4.25:100,000 births (95% CI 1.835–8.377), consistent with the high prevalence recently observed by Broides et al. (7). As this was the first year where such extended and accurate data were available, the true incidence should be re-evaluated in the coming years. There was a predominance of patients of Arab-Muslim decent among our SCID patients (7/8). Genetic founder effects were observed for mutations in *DCLRE1C* and *IL7r*α, which may allow incorporating these mutations into the Israeli Carrier Genetic Screening program for couples of Arab-Muslim decent (25). The high incidence of autosomal recessive SCID in Israel is in contrast with the known high incidence of X-linked SCID (the common γ chain deficiency) in most of the world, and is clearly attributable to the high rate of consanguine marriages and founder genetic effects in our area.

### Confirmation Testing

Currently, the Israeli confirmation algorithm includes a broad range of assays, namely TREC measurement in peripheral blood, proliferation in response to mitogen stimuli and flow cytometry analysis for total lymphocytes, CD3^+^, CD4^+^, and TCR repertoire. In California and Wisconsin, confirmatory tests include CBC and lymphocyte profile only (11, 20), and in New York, mitogen stimulation is performed for select cases (26). In some cases, identification of maternal T cells by various means is needed to diagnose patients with SCID and relatively normal T cell counts including maternally engrafted cells (27). To better evaluate the optimal confirmation algorithm, we have used the extended panel of tests listed above for the duration of the first year of the NBS program. Given the overlapping confidence intervals for the various assays, conclusions on performance data should be treated with caution. Unsurprisingly, TREC in peripheral blood is the most sensitive, but least specific confirmation assay. Mitogen stimulation test and TCR repertoire were the most specific assays in our cohort but not sensitive, unable to identify (by themselves) several SCID patients. Overall, lymphocyte subset analysis (total lymphocytes, CD3^+^, CD4^+^, and CD8^+^) were of satisfactory specificity to be used as standalone confirmation tests. In our cohort, lack of CD20^+^cells was also very informative to identify an underlying genetic defect that is associated with such immune phenotype (such as in *DCLRE1C*). It is important to remember, also, that TCR repertoire analysis could be of great value when lymphocyte profiling is inconclusive and clinical entities such as Omenn’s syndrome or SCID with maternal engraftment are suspected (typical rash, lymphadenopathy, alopecia, etc.). Since the TCR repertoire test is not routinely available in many labs and the TREC assay by itself is known to be very sensitive also in diagnosing patients with these clinical phenotypes (28), we suggest an initial confirmatory panel that will include complete blood count, full lymphocyte profile and TREC quantification in the peripheral blood.

### Secondary Targets

The secondary target of the screening, detection of newborns with T lymphopenia due to non-SCID etiologies, poses several issues that warrant consideration. First, the management of children with genetic syndromes accompanied by lymphopenia, extreme preterms or patients with idiopathic T cell lymphopenia is very often unaffected by a positive NBS result. Many of them recover spontaneously (11). A recent study by Albin-Leeds et al. (29) has reported that while additional infants with T cell lymphopenia are identified in NBS panels, they seem to do well clinically. Children with severe genetic syndromes often present other, more urgent symptoms at birth. Several of these children detected as secondary targets in our cohort were designated by their families as DNR (do not resuscitate), prohibiting any interventions even if medically warranted. Extreme preterms are hospitalized and closely monitored regardless of their screening results, and are often treated as immunocompromised even in the absence of lymphopenia. The same applies for newborns with secondary immunodeficiency as a result of chylothorax, maternal immune suppression, or other causes. Second, while early detection of syndromes with primary immunodeficiencies, such as DiGeorge syndrome, is possible through the NBS program, most newborns with these syndromes have TREC birth levels above the threshold. Barry et al. retrospectively examined NBS results of 1,350 DiGeorge syndrome and found positive screening results in only 11. Five out of these 11 would have been diagnosed with DiGeorge syndrome without NBS (30). Thus, reliance on the screening for secondary targets would be problematic. Nevertheless, because of its well-known relevancy to immunodeficiency, DiGeorge syndrome should be considered in every non-SCID newborn with a positive screening result, even if the typically associated features are absent.

Further studies are required to assess the efficacy and cost–benefit of screening for Non-SCID T cell lymphopenia. Active screening for secondary targets should be pursued if one has sufficient resources to properly workup these children, while keeping in mind that the majority of children with mild T cell abnormalities will return a negative NBS result.

### Considerations Based on Gestational Age/Birth Weight

As expected, TREC correlates to both gestational age and birth weight. However, there is great variability in TREC results between newborns of similar birth age, newborns of similar birth weight and even twins, resulting in only a weak correlation between these parameters and TREC. Nevertheless, several important observations arise from the data.

Both gestational age and birth weight correlate with low TREC in extreme preterms (<28 weeks). This is compounded further by a slower recovery rate in preterms, with infants born 26 weeks gestation or younger in our cohort exhibiting a recovery rate of below four TREC copies/blood spot per week. Thus, for extreme preterms, a lower cutoff or a lenient confirmation approach (whereby extreme preterms are referred to confirmation only if still positive at 37 weeks corrected age), is acceptable (26).

In both our cohort and previously published results by Barbaro et al., TREC copy numbers/blood spot rise significantly over a 2-week period between 28 and 30 weeks gestation (18). Though week-specific data are unavailable, de Felipe et al. describe a similar surge between newborns of 29–31 and 32–36 weeks of gestation (19). This “leap” in TREC increase, preceded and followed by milder, incremental increases, could signal an important maturational period in T cell development. Pragmatically, these data suggest that while a lenient approach toward extreme preterms with positive screening results is acceptable, moderate to late preterms should be screened under the same scrutiny as term infants.

Further studies and analysis of relation between gestational age, birth weight, and TREC are required. As data from similar studies are aggregated, it may be possible to comprise a normal distribution chart for TREC based on gestational age and birth weight, in order to minimize FP results while allowing for prompt assessment of true positives.

### Concluding Remarks

The purpose of NBS is to enable early diagnosis and treatment of life-threatening conditions. As reported above, seven of the eight SCID patients diagnosed through the Israeli screening program in its first year are currently alive and well, and are either post- or awaiting BMT. Before the initiation of the NBS program, such positive clinical outcomes were impossible for children with SCID.

## Author Contributions

ER analyzed the data and wrote the paper; AL and AS performed all confirmatory and genetic tests and analyzed the results; SD, TS-L, and SA performed the screening for TREC and beta-actin and collected the results; TS, AB, AN, NM, OT, SH, ID, PS, and RS followed the patients; AE and RS analyzed the immunological results and supervised the writing of the paper. All authors approved the final version for submission and are accountable for all aspects of the work.

## Conflict of Interest Statement

The authors declare that the research was conducted in the absence of any commercial or financial relationships that could be construed as a potential conflict of interest.
