# Supplementary material for: First Year of Israeli Newborn Screening for Severe Combined Immunodeficiency—Clinical Achievements and Insights
Source: Front Immunol. 2017 Nov 6;8:1448. doi: 10.3389/fimmu.2017.01448 (PMC5682633; doi:10.3389/fimmu.2017.01448)
Supplement: Supplementary file 1 [file data_sheet_1.docx]

Supplementary Material

**First Year of Israeli Newborn Screening for SCID – Clinical Achievements and Insights**

Erez Rechavi^1^, Atar Lev^1^, Amos J Simon^1^, Tali Stauber^1^, Suha Daas^2^, Talia Saraf-Levy^2^, Arnon Broides^3,9^, Amit Nahum^3,9^, Nufar Marcus^4,9^, Suhair Hanna^5,9^, Polina Stepensky^6,9^, Ori Toker^7,9^, Ilan Dalal^8,9^, Amos Etzioni^5,9*^, Shlomo Almashanu^2,*^ and Raz Somech^1,9,10*^

*** Correspondence:** Raz Somech, Raz.Somech@sheba.health.gov.il


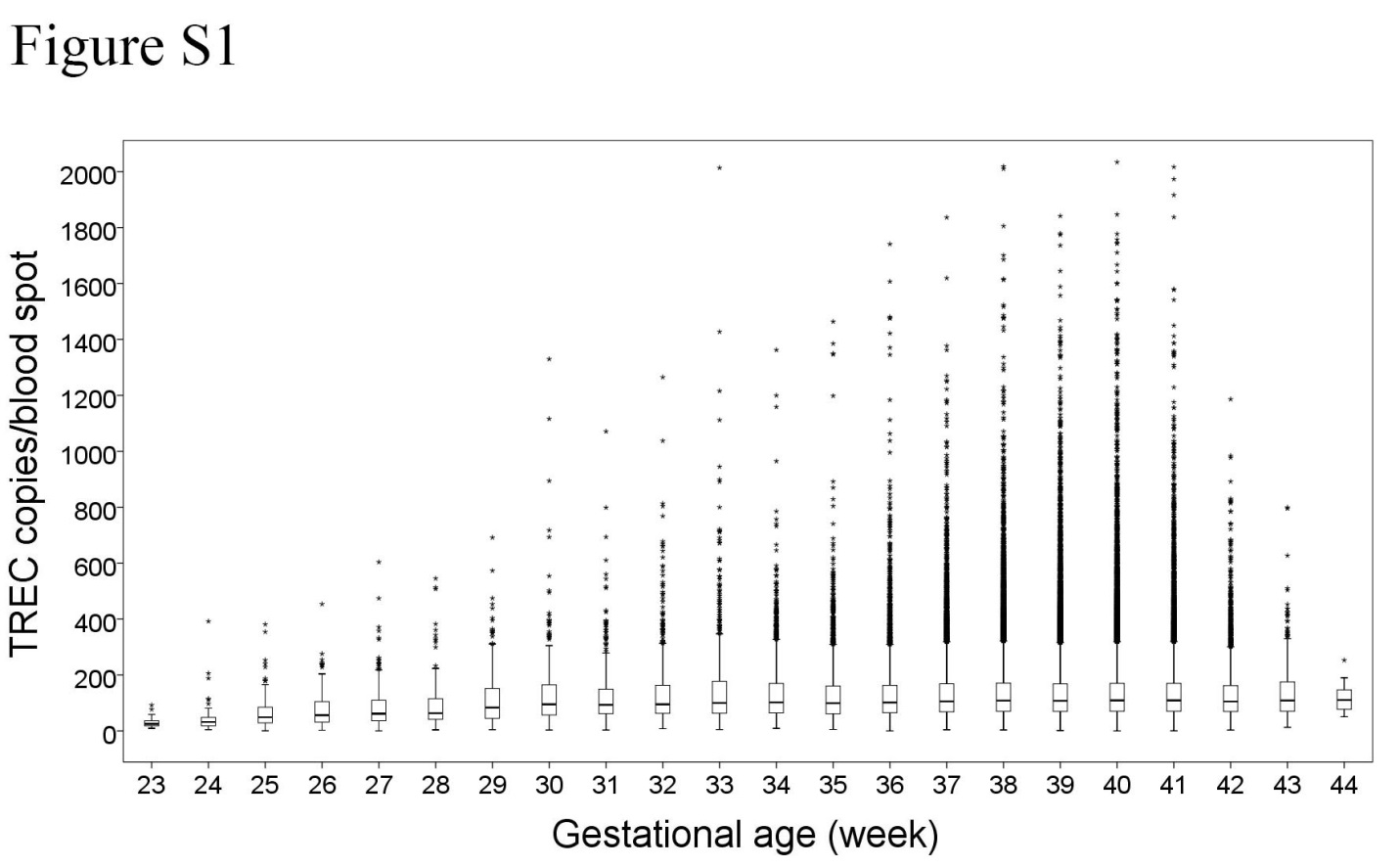


**Supplementary Figure 1.** Box and whiskers plot showing TREC levels for each gestational week. Box = 25^th^ and 75^th^ percentiles, whiskers = 1.5 times height of box or, if no case has a value in that range, the min/max value, asterisks = outliers.


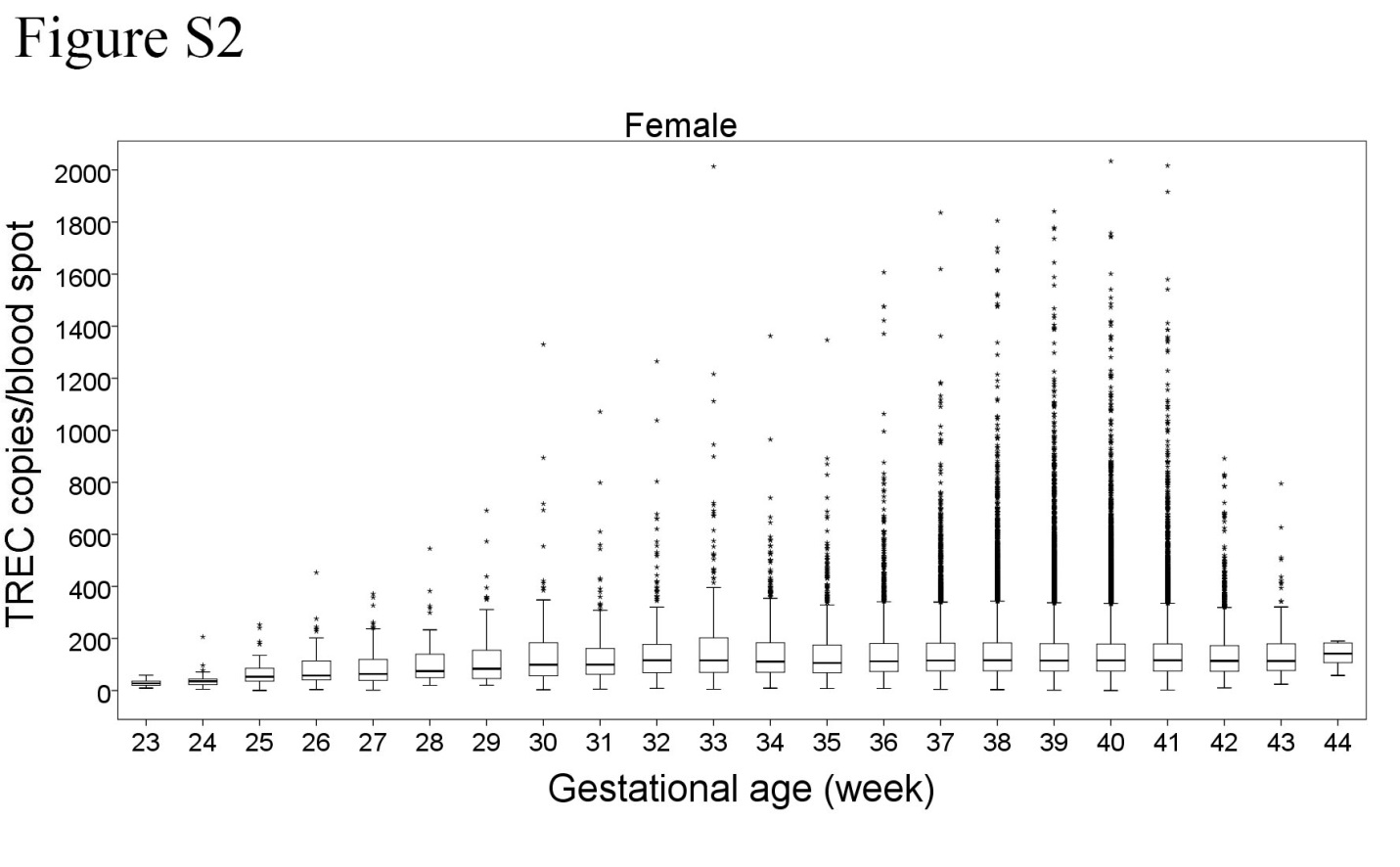

**Supplementary Figure 2.** Box and whiskers plot showing TREC levels for each gestational week for female newborns. Box = 25^th^ and 75^th^ percentiles, whiskers = 1.5 times height of box or, if no case has a value in that range, the min/max value, asterisks = outliers.


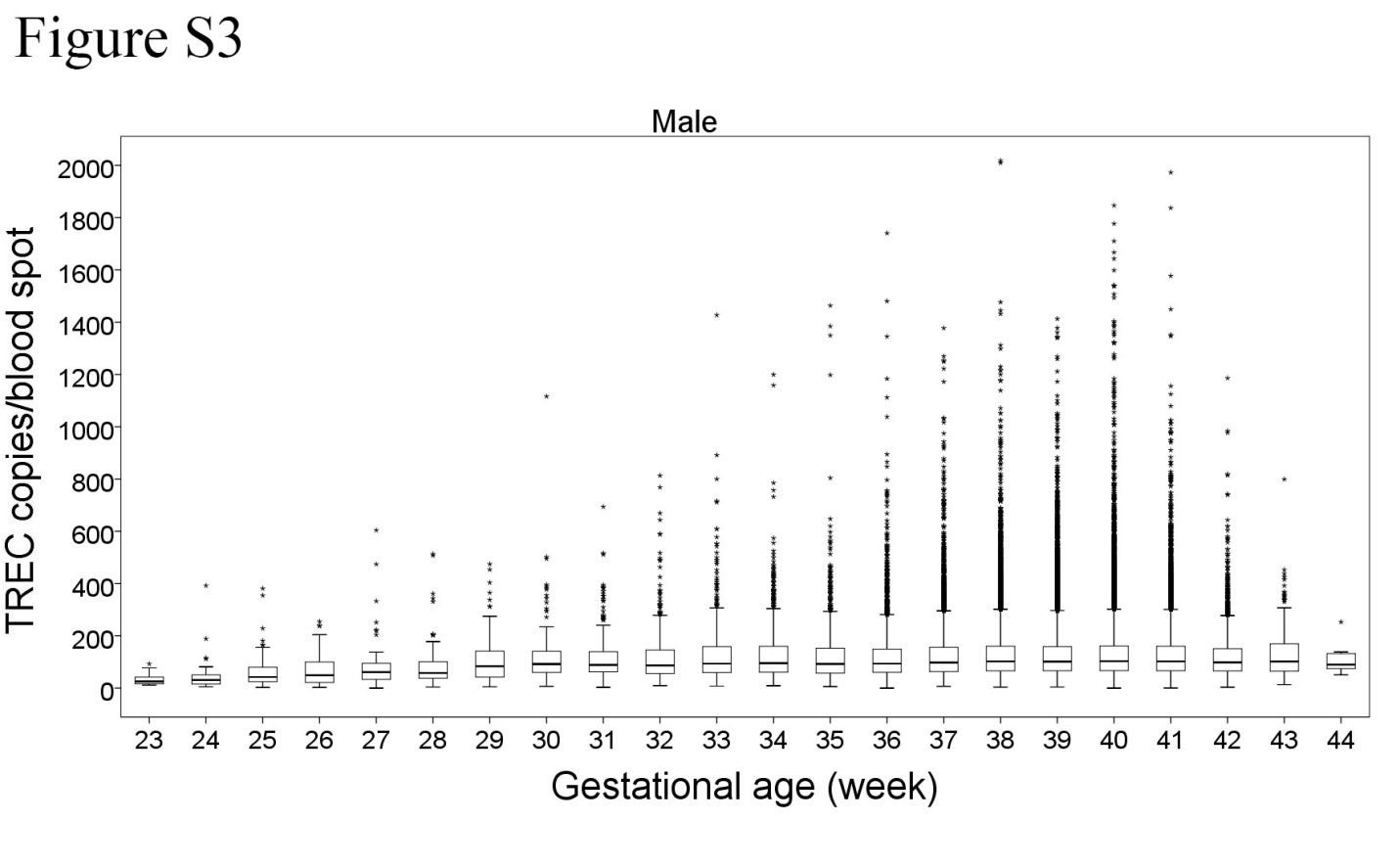


**Supplementary Figure 3.** Box and whiskers plot showing TREC levels for each gestational week for male newborns. Box = 25^th^ and 75^th^ percentiles, whiskers = 1.5 times height of box or, if no case has a value in that range, the min/max value, asterisks = outliers.


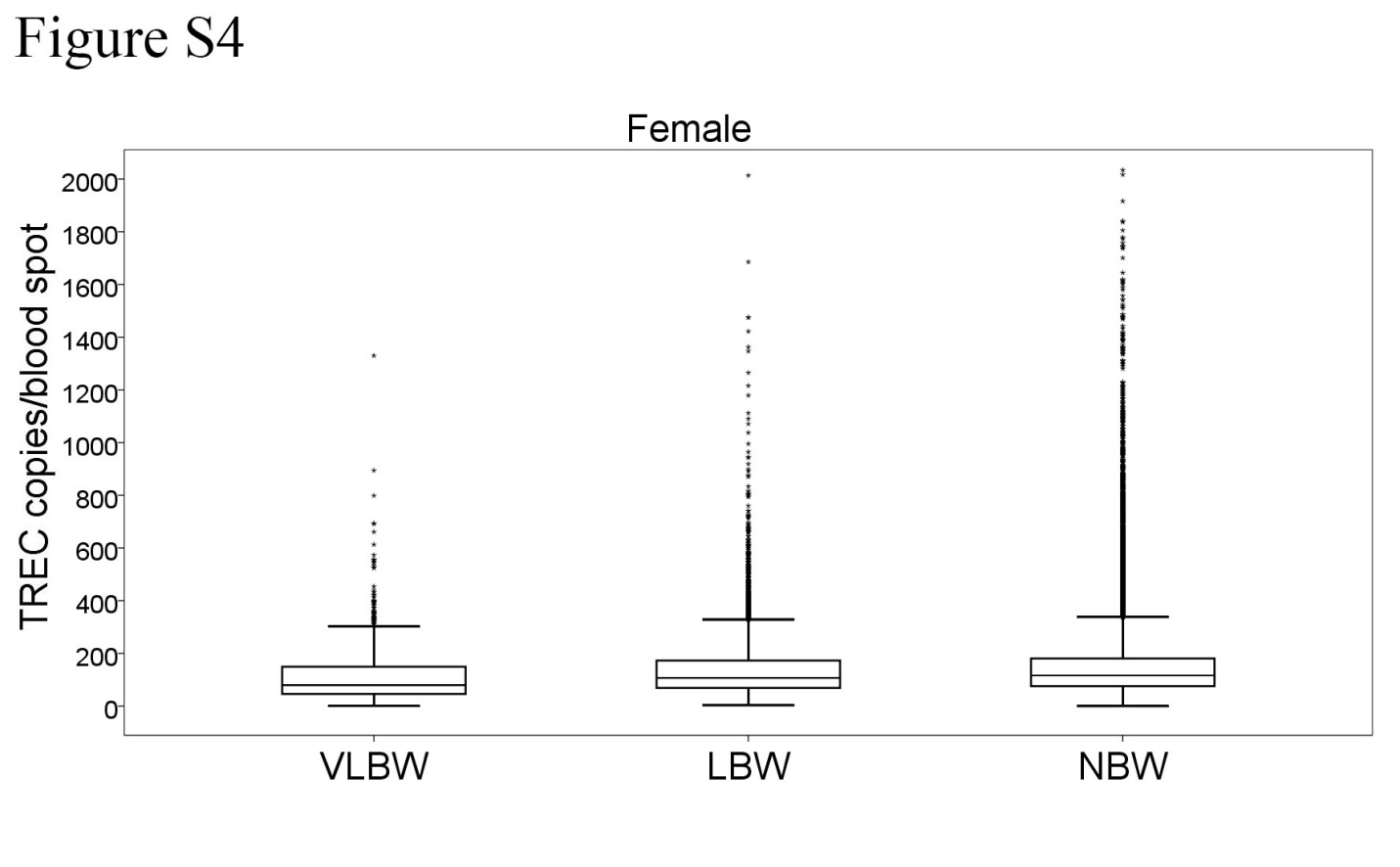


**Supplementary Figure 4.** Box and whiskers plot showing TREC levels for each birth weight group for female newborns. VLBW = Very low birth weight, below 1,500 gr; LBW = Low birth weight, 1,500-2,500 gr; NBW = Normal birth weight, above 2,500 gr. Box = 25^th^ and 75^th^ percentiles, whiskers = 1.5 times height of box or, if no case has a value in that range, the min/max value, asterisks = outliers.


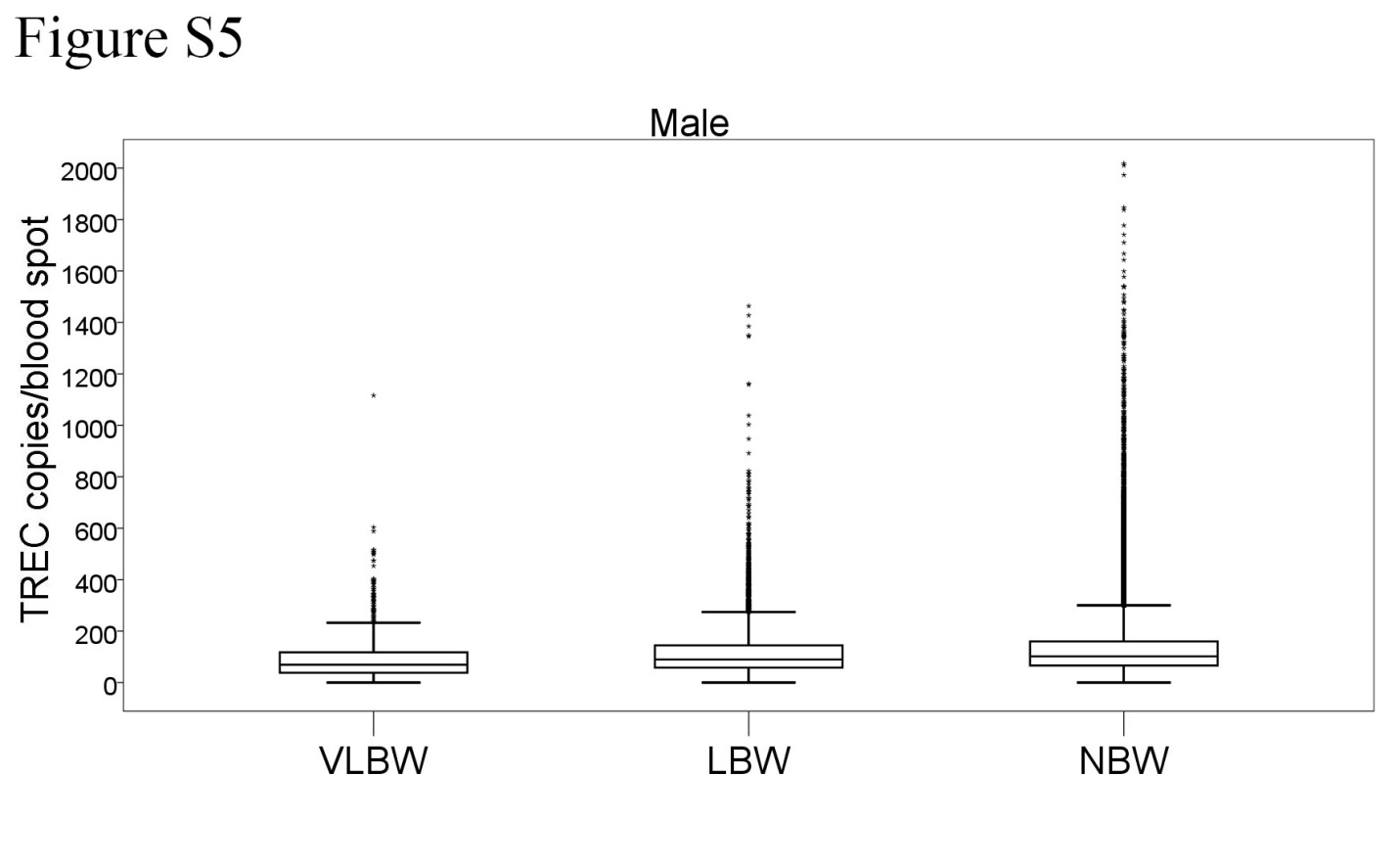


**Supplementary Figure 5.** Box and whiskers plot showing TREC levels for each birth weight group for male newborns. VLBW = Very low birth weight, below 1,500 gr; LBW = Low birth weight, 1,500-2,500 gr; NBW = Normal birth weight, above 2,500 gr. Box = 25^th^ and 75^th^ percentiles, whiskers = 1.5 times height of box or, if no case has a value in that range, the min/max value, asterisks = outliers.
